# Transcriptional regulators *SP110* and *SP140* modulate inflammatory response genes in *Mycobacterium tuberculosis*-infected human macrophages

**DOI:** 10.1128/spectrum.00101-24

**Published:** 2024-08-20

**Authors:** Hajime Nakamura, Haruka Hikichi, Shintaro Seto, Minako Hijikata, Naoto Keicho

**Affiliations:** 1 Department of Pathophysiology and Host Defense, The Research Institute of Tuberculosis, Japan Anti-Tuberculosis Association, Tokyo, Japan; 2 Department of Basic Mycobacteriosis, Nagasaki University Graduate School of Biomedical Sciences, Nagasaki, Japan; 3 The Research Institute of Tuberculosis, Japan Anti-Tuberculosis Association, Tokyo, Japan; Geisel School of Medicine at Dartmouth, Lebanon, New Hampshire, USA

**Keywords:** *Mycobacterium tuberculosis*, macrophage, transcriptional factor, *SP110*, *SP140*, RNA sequencing, interferon response, oxidative phosphorylation

## Abstract

Understanding the functions of human transcriptional regulatory genes *SP110* and *SP140* during *Mycobacterium tuberculosis* infection is crucial; in a mouse model, homologous genes *Sp110* and *Sp140* have been shown to negatively regulate inflammatory response genes, including the type I interferon (IFN) response. The reduction of these genes in mice is associated with susceptibility to *M. tuberculosis* infection and the development of necrotizing granulomatous lesions. To investigate the involvement of *SP110* and *SP140* in human inflammatory response, we analyzed their regulatory manner in THP-1 macrophages infected with *M. tuberculosis*. Genome-wide transcriptional profiling revealed that the depletion of *SP110* and/or *SP140* impaired the induction of gene expression associated with inflammatory responses, including IFN response genes, although it had little effect on the intracellular proliferation of *M. tuberculosis*. By contrast, genes related to phosphorylation were upregulated in infected macrophages with *SP110* and/or *SP140* knockdown, but downregulated in infected control macrophages without their knockdown. Reverse transcription-quantitative PCR and ELISA further confirmed the impairment of the induction of IFN response genes by the depletion of *SP110* and/or *SP140* in *M. tuberculosis*-infected macrophages. These findings suggest that human *SP110* and *SP140* act as positive regulators for genes associated with inflammatory responses in *M. tuberculosis*-infected macrophages.

**IMPORTANCE:**

Tuberculosis (TB) is one of the most serious infectious diseases, with high morbidity and mortality worldwide. C3HeB/FeJ mice are widely utilized for evaluating anti-TB drugs because their drug sensitivity and pathology during *M. tuberculosis* infection resemble those of human TB, including the development of necrotizing granulomas. Downregulation of the transcriptional regulatory genes *Sp110* and *Sp140* in C3HeB/FeJ mice has been demonstrated to activate gene expression associated with inflammatory responses during *M. tuberculosis* infection, resulting in susceptibility to the infection. Here, we examined the regulatory manner of *SP110* and *SP140* using transcriptomic analysis in *M. tuberculosis*-infected human macrophages. Depletion of *SP110* and/or *SP140* in *M. tuberculosis*-infected THP-1 macrophages impaired the induction of gene expression associated with inflammatory responses, including interferon response genes, compared with that in control macrophages. These results suggest that human *SP110* and *SP140* act as positive regulators for genes associated with inflammatory responses upon *M. tuberculosis* infection.

## INTRODUCTION

Tuberculosis (TB) is a chronic inflammatory disease caused by *Mycobacterium tuberculosis* infection, with high morbidity and mortality worldwide. A total of 1.3 million people died from TB in 2022. In addition, the number of newly diagnosed TB cases increased to 7.5 million in 2022 from 6.4 million in 2021 and 5.8 million in 2020, reflecting the impact of the COVID-19 pandemic (1).

Infection with *M. tuberculosis* leads to the development of granulomatous lesions in the affected organisms. Despite the high heterogeneity of granulomas, necrotizing granulomatous lesions are a hallmark of TB pathology (2, 3). The environment at the center of necrotizing granulomas, including acidic pH, low oxygen tension, and limited nutrients, contributes to reducing the burden of infected *M. tuberculosis*. However, a small population survives and shifts to a nonreplicating persistence state within granulomas (4). The remaining persistent bacteria enter a dormant state, causing latent TB infection (LTBI). Depending on the host’s immune status, active TB can develop from LTBI. Long-term chemotherapy is required to eradicate *M. tuberculosis* infection in patients with TB. Failure or inadequate chemotherapy causes selective growth of drug-resistant mutants, resulting in the development of acquired drug-resistant TB.

Standard laboratory mouse strains, such as C57BL/6 and BALB/c, have been most widely used in TB drug development (5). However, these mouse strains exhibit a single lesion type during *M. tuberculosis* infection, which is not the necrotizing granulomas. Kramnik et al. reported that infection with *M. tuberculosis* develops necrotizing granulomatous lesions in the lungs of C3HeB/FeJ mice, followed by further reports evaluating their histopathology and pathogenicity in detail (6–10). It has been shown that the efficacy of anti-TB drugs in C3HeB/FeJ mice infected with *M. tuberculosis* resembles that in patients with TB because of developing necrotizing granulomatous lesions (11). This mouse model is now widely employed for evaluating new anti-TB drugs and regimens (12).

Host genetic analysis has identified that the *super susceptibility to tuberculosis 1* (*Sst1*) locus is associated with susceptibility to *M. tuberculosis* infection in C3HeB/FeJ mice (13). Further analyses demonstrated that impaired gene expression of *Sp110* and *Sp140* in the *Sst1* locus results in inflammatory activation *via* the type I interferon (IFN) pathway, causing susceptibility to *M. tuberculosis* infection and development of necrotizing granulomas within the mouse lungs (14, 15). Recently, the *Sp140* gene is responsible for activation of the type I IFN pathway and susceptibility to *M. tuberculosis* infection in mice (16). However, necrotizing granulomatous lesions do develop in the human lungs, even when *SP110* and *SP140* genes are fully expressed in immune cells. This discrepancy suggests that the response of these genes to *M. tuberculosis* infection differs between humans and mice.

In this study, we explored the regulatory manner of *SP110* and/or *SP140* in the human macrophage cell line, THP-1, infected with *M. tuberculosis*. Because both genes act as transcriptional regulators (17), we conducted the knockdown of their gene expression in *M. tuberculosis*-infected macrophages and surveyed genome-wide transcriptional profiles. Knockdown of *SP110* and/or *SP140* impaired the induction of gene expression associated with inflammatory responses, including IFN response genes, in *M. tuberculosis*-infected macrophages. These results suggest that human *SP110* and *SP140* act rather as positive regulators for genes associated with inflammatory responses upon *M. tuberculosis* infection.

## RESULTS

### Depletion of *SP110* and/or *SP140* expression in THP-1 macrophages

**Fig 1:**
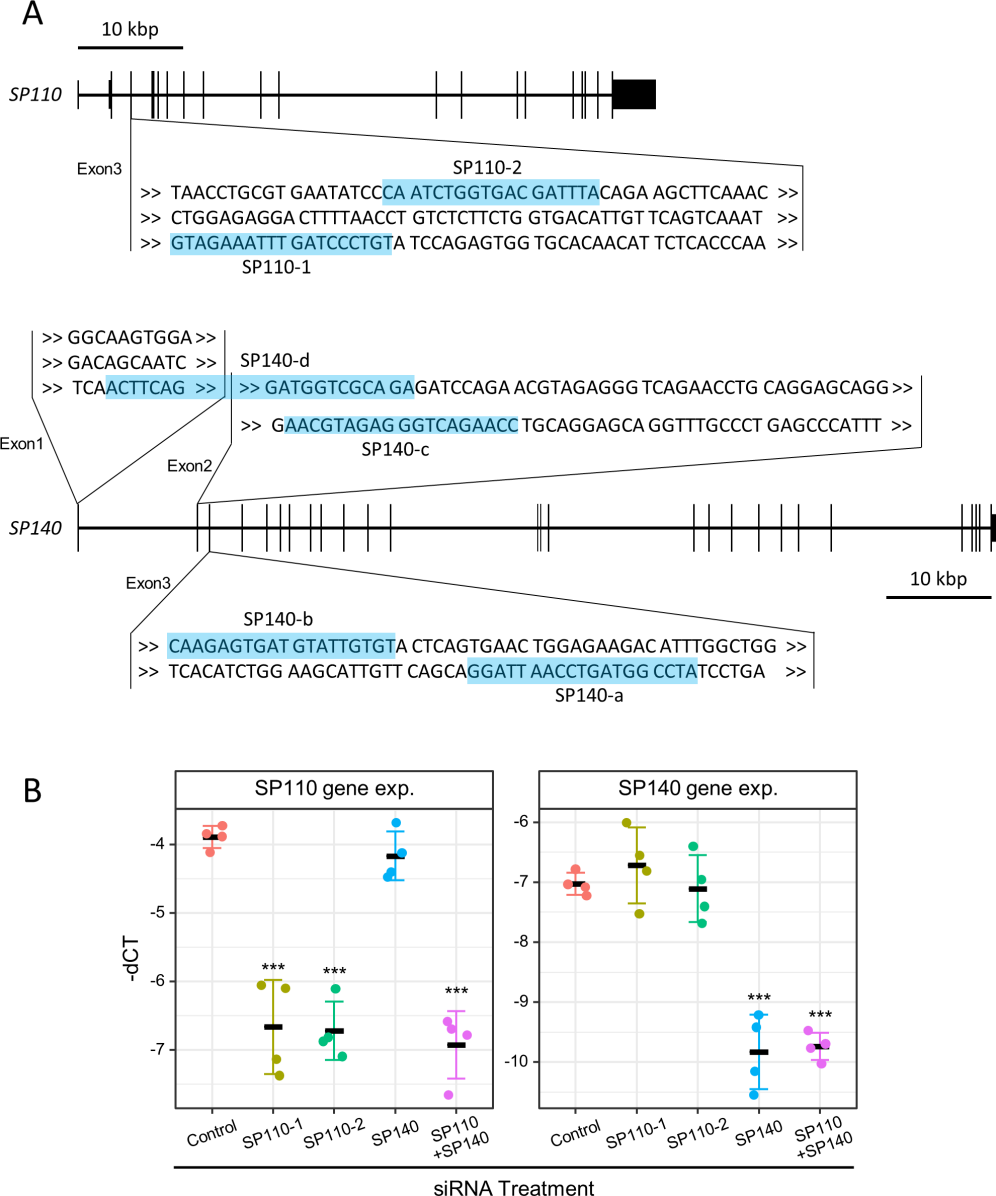
Knockdown of *SP110* and/or *SP140* by siRNA treatment in THP-1 macrophages. (**A**) Design of siRNA targeting *SP110* and *SP140*. Exon sequences targeted by siRNA molecules are highlighted. (**B**) Knockdown efficacy of *SP110* and/or *SP140* genes. PMA-stimulated THP-1 cells were treated with siRNA targeting *SP110* and/or *SP140*. The expression levels of *SP110* and *SP140* in transfected cells were investigated by RT-qPCR (*n* = 4). Two molecules were used for siRNA targeting *SP110* (SP110-1 and SP110-2). Four molecules were mixed and used for siRNA targeting *SP140* (SP140). SP110-1 and SP140 were mixed and used for knockdown targeting *SP110* and *SP140* (SP110−1 + SP140). The values of –dCT are shown as gene expression (gene exp.). ***P < 0.001, as determined by ANOVA with Dunnett’s test.

We have demonstrated that infection with *M. tuberculosis* upregulates the gene expression related to IFN responses in phorbol myristate acetate (PMA)-stimulated THP-1 cells (18), as described previously (19). To explore the involvement of *SP110* and *SP140* in gene expression, including IFN response genes, in *M. tuberculosis*-infected macrophages, we employed small interfering RNA (siRNA) molecules targeting *SP110* and *SP140* to deplete their expression in THP-1 macrophages. We designed two sets of siRNA molecules for depleting *SP110* or *SP140* and verified the knockdown efficacy by reverse transcription-quantitative PCR (RT-qPCR) (Fig. 1A). The designed siRNA molecules for *SP110* decreased the expression of *SP110* significantly but had a slight effect on the expression of *SP140* (Fig. 1B). Conversely, one siRNA molecule designed for *SP140* decreased the expression of both *SP110* and *SP140*, and the other did not decrease *SP140* expression (Fig. S1). Next, we employed a mixture containing four different siRNA molecules targeting *SP140* (Fig. 1A) as described previously (20). Using this mixture of siRNA molecules against *SP140*, we observed a significant decrease in *SP140* expression without affecting *SP110* expression (Fig. 1B). To deplete the expression of both *SP110* and *SP140*, we treated THP-1 macrophages with the combination of a single siRNA molecule targeting *SP110* and a mixture of siRNA molecules targeting *SP140*, confirming the reduction in the expression of both genes. We verified the reduction of SP110 protein expression in THP-1 macrophages by siRNA treatment against the *SP110* gene (Fig. S2), despite the inability to detect SP140 protein with the available antibodies.

### Intracellular proliferation of *M. tuberculosis* and cell viability of *SP110*- and/or *SP140*-knockdown macrophages

**Fig 2:**
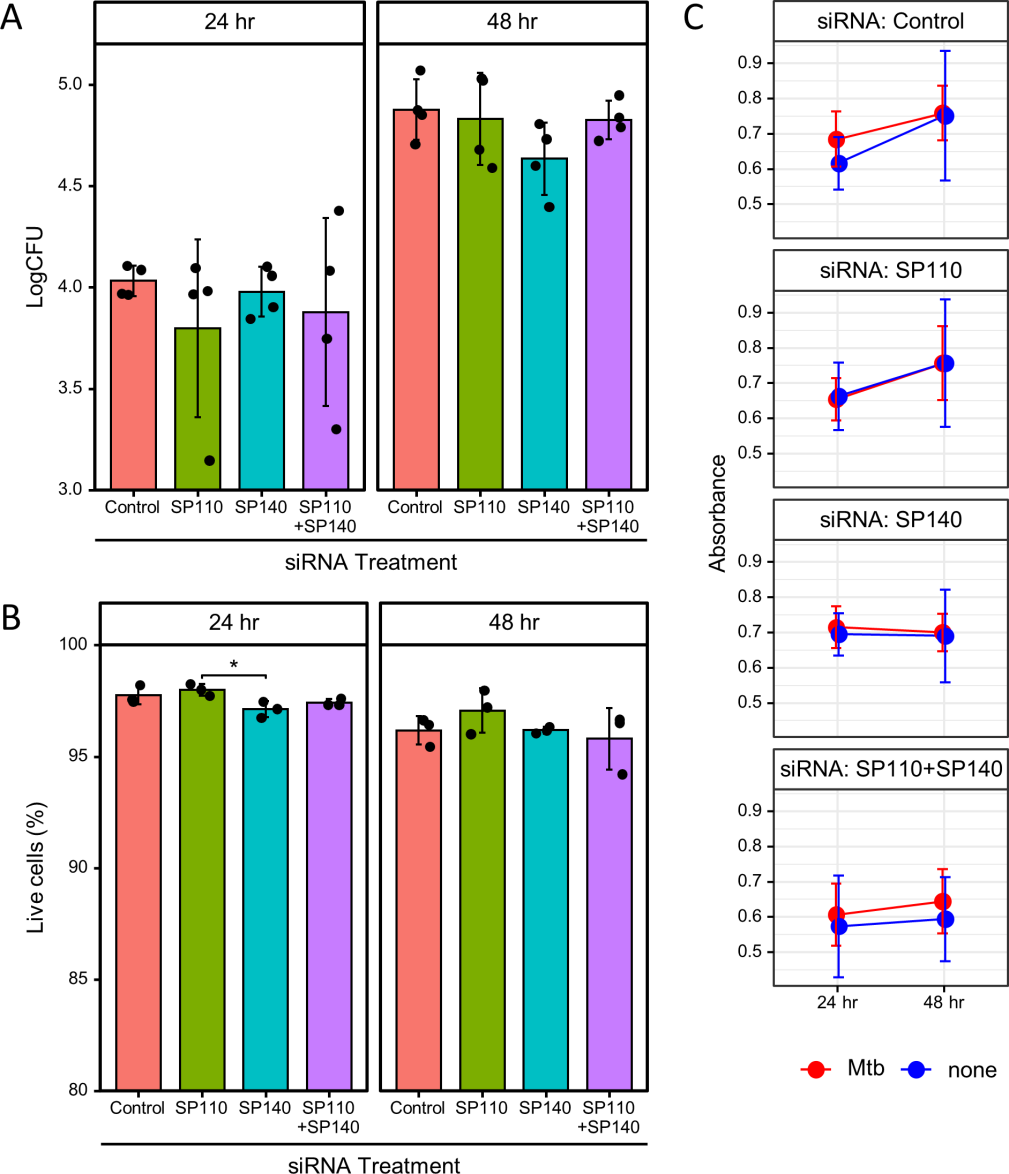
Intracellular proliferation of *M. tuberculosis* and viability of infected macrophages treated with siRNA targeting *SP110* and/or *SP140*. PMA-stimulated THP-1 cells were treated with siRNA targeting *SP110* and/or *SP140*, followed by infection with *M. tuberculosis*. (**A**) Intracellular proliferation of *M. tuberculosis* within macrophages. *M. tuberculosis*-infected macrophages treated with siRNA targeting the indicated genes were harvested at 24 and 48 h post-infection (p.i.). The values of bacterial numbers were determined by CFU assays. Mean and SD values of bacterial numbers are also presented from four independent experiments (*n* = 4). (**B**) Viability of macrophages. The viability of *M. tuberculosis*-infected macrophages treated with siRNA targeting the indicated genes was measured using a LIVE/DEAD fixable stain. Mean and SD values of the proportion of live cells are presented (*n* = 3). *: *P* < 0.05, as determined by ANOVA with Tukey–Kramer multiple comparison test. (**C**) Growth activity of macrophages. The growth activity of uninfected and *M. tuberculosis*-infected macrophages treated with siRNA targeting the indicated genes was measured by MTT assay. Mean and SD values of the optical absorbance are presented (*n* = 6).

We evaluated the intracellular proliferation of *M. tuberculosis* within *SP110*- and/or *SP140*-knockdown macrophages (Fig. 2A). Since the gene expression of the target genes for siRNA treatment was increased to approximately 30%–70% of that in control macrophages 5 days post-transfection under our experimental conditions, we examined the intracellular proliferation of *M. tuberculosis* at 24 or 48 h post-infection (p.i.), corresponding to three or 4 days post-transfection, respectively. At 24 and 48 h p.i., the expression of *SP110* and/or *SP140* significantly decreased by the treatment with siRNA against target genes in *M. tuberculosis*-infected macrophages (Fig. S3). We observed no significant differences in the intracellular proliferation between control macrophages and *SP110*- and/or *SP140*-knockdown macrophages.

We next investigated the induction of cell death and inhibition of cell growth in *SP110-* and/or *SP140*-knockdown macrophages infected with *M. tuberculosis* (Fig. 2B and C). Both flow cytometric analysis using LIVE/DEAD dye-stained cells and the MTT assay revealed that knockdown of *SP110* and/or *SP140* did not significantly induce cell death or inhibit growth in *M. tuberculosis*-infected macrophages.

We further measured the concentration of ATP in THP-1 macrophages with siRNA treatment against *SP110* and/or *SP140* (Fig. S4). *M. tuberculosis* infection decreased the concentration of ATP in macrophages, whereas siRNA treatment against *SP110* and/or *SP140* did not change the concentration of ATP in macrophages. These results demonstrated that the knockdown of *SP110* and/or *SP140* did not change cell viability, growth rate, and ATP concentration in *M. tuberculosis*-infected macrophages in the early infection period.

### Genome-wide transcriptional profiling in *SP110*- and/or *SP140*-knockdown macrophages infected with *M. tuberculosis*

**Fig 3:**
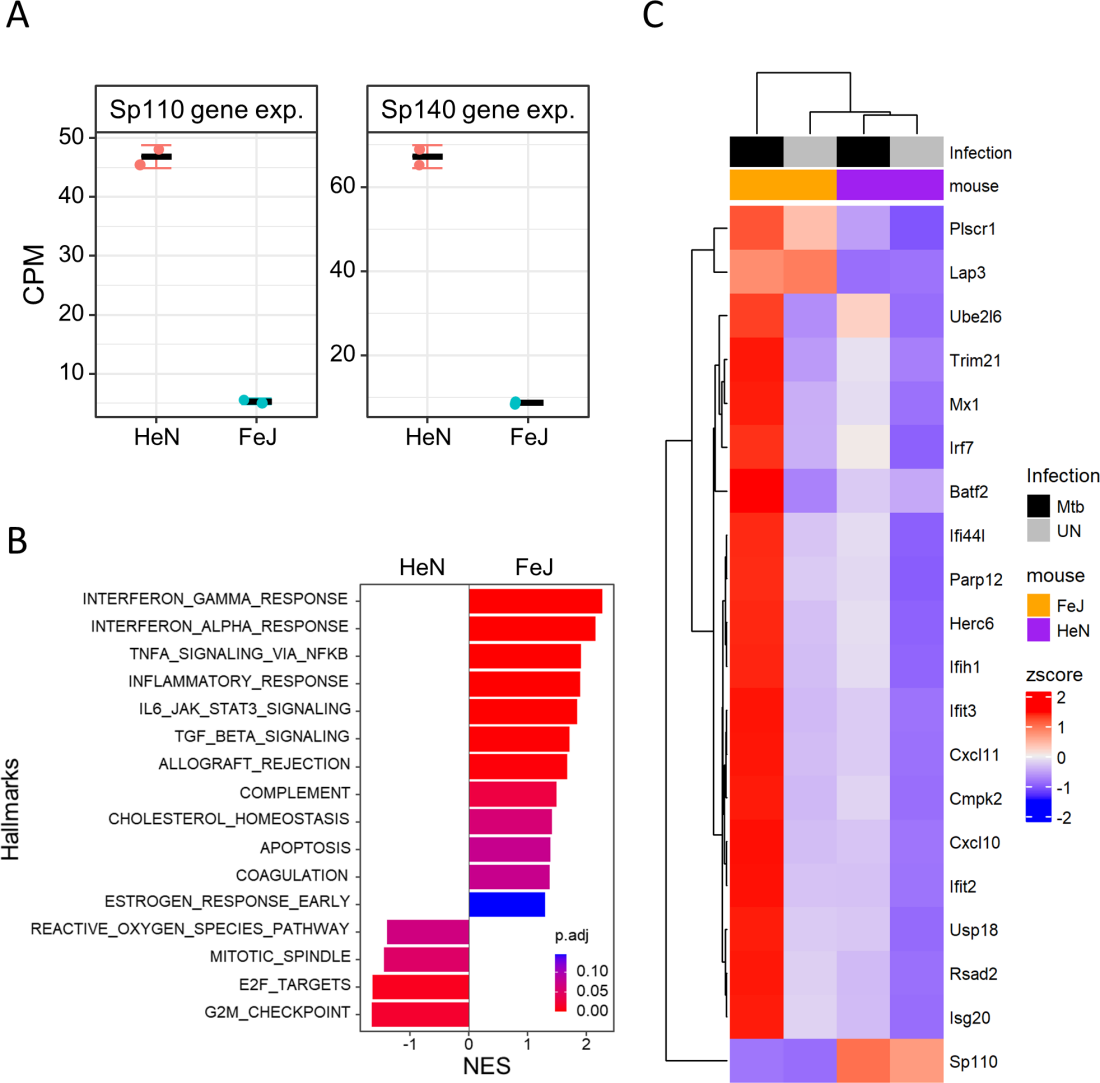
Genome-wide analysis of the gene expression profiles of *M. tuberculosis*-infected macrophages derived from C3HeB/FeJ and CH/HeN mice. (**A**) Gene expression of *Sp110* and *Sp140* in BMMs. Count per million (CPM) of *Sp110* and *Sp140* genes in BMMs from C3HeB/FeJ (FeJ) and C3H/HeN (HeN) mice are indicated. (**B**) Comparison of gene expression in *M. tuberculosis*-infected BMMs between C3HeB/FeJ and C3H/HeN mice. BMMs from FeJ and HeN mice were infected with *M. tuberculosis* for 24 h (*n* = 2). RNA was extracted and subjected to messenger RNA sequencing (mRNA-seq). Gene set enrichment analysis (GSEA) was conducted on *M. tuberculosis*-infected BMMs from C3HeB/FeJ (FeJ) and C3H/HeN (HeN) mice. Significantly enriched hallmarks are depicted, representing both activated and suppressed hallmarks in *M. tuberculosis*-infected BMMs from C3HeB/FeJ mice compared with those from C3H/HeN mice. The bar size indicates the normalized enrichment score (NES) for activated and repressed hallmarks in FeJ mice. The color scale indicates the adjusted *P* value. NES, normalized enrichment score. *P*. adjust, adjusted *P* values. (**C**) Heatmap visualization of the expression of IFN response genes. The major genes were obtained from the enriched GSEA hallmark gene sets of IFN-alpha and IFN-gamma responses. The top 20 genes were selected using adjusted *P* values, followed by hierarchical clustering analysis. Annotation bars indicate infection with *M. tuberculosis* and BMMs from FeJ or HeN mice.

Previous studies have shown that *M. tuberculosis* infection enhances the expression of inflammatory genes, including type I IFN response genes, in lungs from *Sst1*-congenic and *Sp140-*knockout mice compared with that in wild-type mice (16, 21). This suggests that *Sp110* and *Sp140* play negative regulatory roles in the inflammatory responses of *M. tuberculosis*-infected mouse lungs. To evaluate the function of *Sp110* and *Sp140* on the gene expression profiles in murine bone marrow-derived macrophages (BMMs) upon *M. tuberculosis* infection, we performed transcriptomic analysis using mRNA sequencing (mRNA-seq) in *M. tuberculosis*-infected BMMs derived from C3HeB/FeJ and C3H/HeN mice (Fig. 3). C3HeB/FeJ shares a common origin with C3H/HeJ, while C3HeB/FeJ carries a normal allele at the *Tlr4* locus unlike C3H/HeJ (https://www.jax.org/strain/000658). Therefore, we used C3H/HeN mice, carrying the normal *Tlr4* allele, as the control. The expression of both *Sp110* and *Sp140* genes in BMMs from C3HeB/FeJ mice was lower than that from C3H/HeN mice, as expected (Fig. 3A). mRNA-seq analysis revealed that the expression of inflammatory genes, including IFN response genes, in *M. tuberculosis*-infected BMMs from C3HeB/FeJ mice was enhanced compared with that of C3H/HeN mice (Fig. 3B and C). This suggests that *Sp110* and/or *Sp140* play negative regulatory roles in the inflammatory responses, including IFN response genes, in *M. tuberculosis*-infected mouse macrophages.

**Fig 4:**
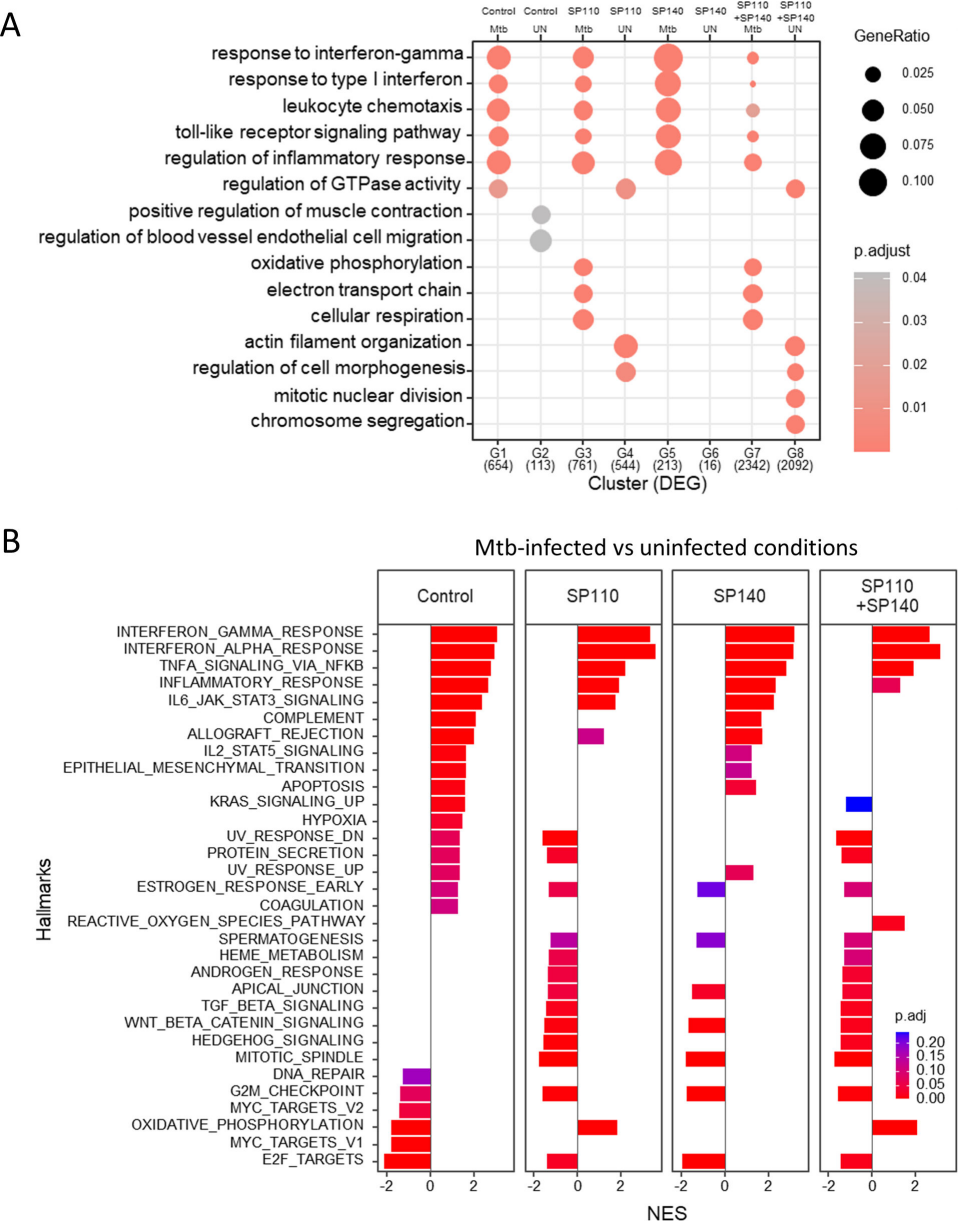
Genome-wide analysis of the gene expression profiles of *M. tuberculosis*-infected macrophages treated with siRNA targeting *SP110* and/or *SP140*. (**A**) GOBP enrichment analysis for macrophages. THP-1 macrophages treated with siRNA against *SP110* and/or *SP140* were infected with *M. tuberculosis* for 24 h (*n* = 5). RNA was extracted and subjected to mRNA-seq. The list includes upregulated (G1, G3, G5, and G7) and downregulated (G2, G4, G6, and G8) DEGs of *M. tuberculosis*-infected macrophages (Mtb) compared with those in uninfected macrophages (UN) treated with siRNA targeting control (G1 and G2), *SP110* (G3 and G4), *SP140* (G5 and G6), or *SP110* and *SP140* (G7 and G8). G1; 654 genes, G2; 113 genes, G3; 761 genes, G4; 544 genes, G5; 213 genes, G6; 16 genes, G7; 2342 genes, and G8; 2092 genes. The significant GOBP terms for upregulated and downregulated DEGs in treated macrophages are shown. GeneRatio, the gene ratio of the indicated GO term in DEG. *P*. adjust, adjusted *P* values. (**B**) GSEA of *M. tuberculosis*-infected macrophages. Significantly enriched hallmarks are shown, representing both activated and suppressed hallmarks in *M. tuberculosis*-infected macrophages compared with those in uninfected macrophages. The bar size indicates the normalized enrichment score (NES) for activated and suppressed hallmarks in infected macrophages. Target genes for siRNA treatment are also indicated. The color scale indicates the adjusted *P* value. NES, normalized enrichment score. p. adjust, adjusted *P* values.

To examine the function of *SP110* and *SP140* in regulating the gene expression associated with inflammatory responses, including IFN response genes, and other signaling pathways in *M. tuberculosis*-infected human macrophages, we performed transcriptomic analysis using mRNA-seq (Fig. 4) in PMA-stimulated THP-1 cells with *SP110* and/or *SP140* knockdown, followed by *M. tuberculosis* infection. We infected THP-1 macrophages treated with siRNA against *SP110* and/or *SP140* with *M. tuberculosis* for 24 h, and subsequently performed RNA extraction and mRNA-seq. We identified the DEGs between infected and uninfected macrophages treated with siRNA and performed subsequent gene ontology for biological process (GOBP) enrichment analysis (Fig. 4A). As reported previously (18), infection with *M. tuberculosis* induced the expression of inflammatory genes, including type I and type II IFN response genes, in all conditions tested. In *SP110*-knockdown macrophages, these genes were also induced by infection with *M. tuberculosis*. In *SP110*-knockdown macrophages, infection with *M. tuberculosis* activated the expression of genes associated with oxidative phosphorylation. The results of gene set enrichment analysis (GSEA) were consistent with those of the GOBP enrichment analysis (Fig. 4B). Genes associated with inflammatory responses, including IFN responses and TNF signaling, were upregulated in infected macrophages, and those associated with oxidative phosphorylation were downregulated in infected control macrophages without their knockdown. In *SP110*- and/or *SP140*-knockdown macrophages, genes associated with inflammatory responses were also upregulated upon infection with *M. tuberculosis*. In *SP110*-knockdown macrophages, genes associated with oxidative phosphorylation were upregulated with infection (Fig. S5).

**Fig 5:**
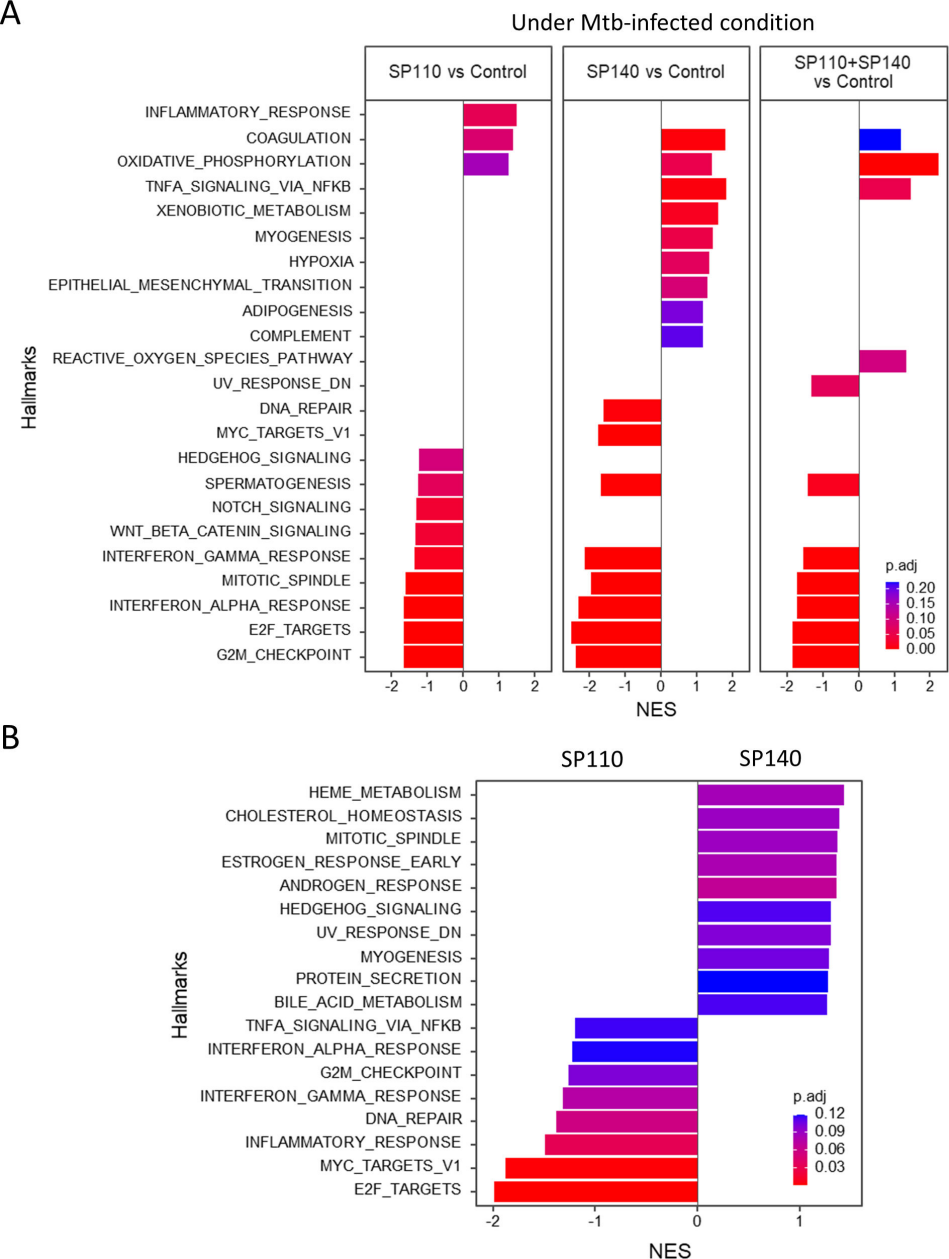
Comparison of gene expression among *M. tuberculosis*-infected macrophages treated with siRNA targeting *SP110* and/or *SP140*. (**A**) GSEA was conducted on *M. tuberculosis*-infected macrophages treated with siRNA targeting control and indicated genes. Significantly enriched hallmarks are depicted, representing both activated and suppressed hallmarks in *M. tuberculosis*-infected macrophages treated with siRNA targeting *SP110* and/or *SP140* compared with those in uninfected macrophages treated with siRNA. (**B**) GSEA of *M. tuberculosis*-infected macrophages treated with siRNA for *SP140* compared with those treated with siRNA for *SP110*. Significantly enriched hallmarks are depicted, representing both activated and suppressed hallmarks in *M. tuberculosis*-infected macrophages treated with siRNA for *SP140* compared with those in *M. tuberculosis*-infected macrophages treated with siRNA for *SP110*. The bar size indicates the NES for activated and repressed hallmarks in *SP110*- and/or *SP140*-knockdown macrophages. The color scale indicates the adjusted *P* value. NES, normalized enrichment score. *P*. adjust, adjusted *P* values.

**Fig 6:**
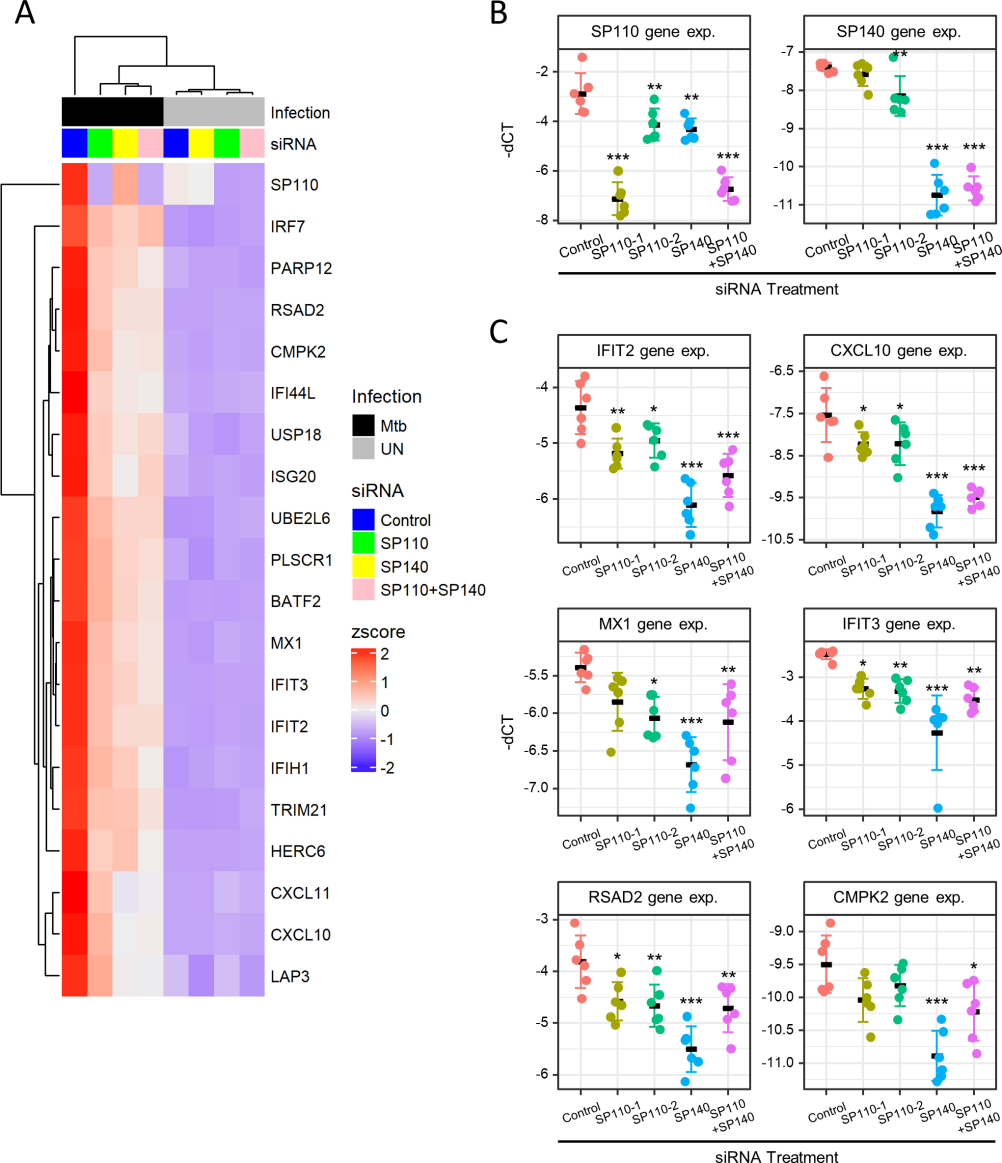
Heatmap visualization and validation of expression in selected IFN response genes. (**A**) Heatmap visualization of the expression of IFN response genes. The major genes were obtained from the enriched GSEA hallmark gene sets of IFN-alpha and IFN-gamma responses. The top 20 genes were selected using adjusted *P* values, followed by hierarchical clustering analysis. Annotation bars indicate infection with *M. tuberculosis* and siRNA treatment conditions. (**B, C**) Gene expression related to IFN response pathways was investigated by RT-qPCR. THP-1 macrophages treated with siRNA against *SP110* and/or *SP140* were infected with *M. tuberculosis* for 24 h (*n* = 6). RNA was extracted and subjected to RT-qPCR. (**B**) *SP110* and *SP140* expression, (**C**) IFN response genes. The values of −dCT are depicted as gene expression (gene exp.). *P < 0.05, **P < 0.01, ***P < 0.001, as determined by ANOVA with Dunnett’s test.

Next, we compared gene expression of *M. tuberculosis-*infected *SP110*- and/or *SP140*-knockdown macrophages with the infected control macrophages using GSEA (Fig. 5A). Infected knockdown macrophages exhibited downregulation of gene expression associated with IFN responses, whereas genes associated with oxidative responses were upregulated. These findings suggest that both *SP110* and *SP140* play a positive regulatory role in IFN responses but a negative regulatory role in oxidative phosphorylation in *M. tuberculosis*-infected human macrophages. To explore deeper into the effect of *SP110* and/or *SP140* knockdown on the expression of genes associated with IFN responses, we extracted the 20 most significant genes for hierarchical clustering (Fig. 6A). For the hallmarks of IFN-alpha and IFN-gamma responses, we identified significant genes shared by the gene sets of both hallmarks. Infection with *M. tuberculosis* significantly enhanced the expression of genes associated with IFN responses in control macrophages. However, these responses were compromised in *SP110*- and/or *SP140*-knockdown macrophages.

We also directly compared the gene expression between infected *SP110*- and *SP140*-knockdown macrophages (Fig. 5B). The expression of genes associated with inflammatory responses, including IFN responses, was repressed in infected *SP140*-knockdown macrophages compared with that in *SP110*-knockdown macrophages. We also investigated the knockdown effect of *SP110* and/or *SP140* in uninfected THP-1 macrophages by GSEA (Fig. S6). Knockdown of *SP110* and/or *SP140* resulted in the induction of gene expression associated with TNF-α signaling pathway and inflammatory response but did not alter the gene expression associated with IFN responses. Collectively, these findings suggest that both *SP110* and *SP140* enhance the expression of genes associated with inflammatory responses, including IFN responses, with *SP140* having a greater impact on these regulations than *SP110* upon *M. tuberculosis* infection in human macrophages.

### Confirmation of mRNA-seq results by RT-qPCR and ELISA

**Fig 7:**
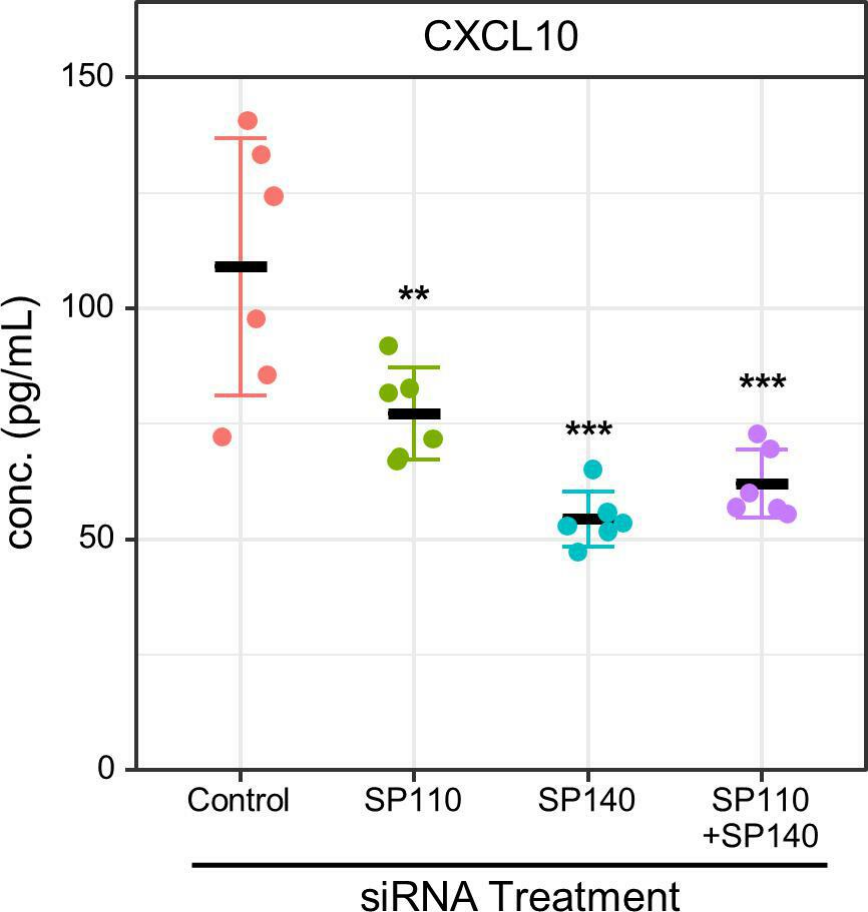
Reduction of CXCL10 secretion by knockdown of *SP110* and/or *SP140*. ELISA for secreted CXCL10 in *M. tuberculosis*-infected THP-1 macrophages with *SP110* and/or *SP140* knockdown. THP-1 macrophages treated with siRNA against *SP110* and/or *SP140* were infected with *M. tuberculosis* for 24 h (*n* = 6). Culture media were collected and subjected to ELISA for CXCL10. **P < 0.01, ***P < 0.001, as determined by ANOVA with Dunnett’s test.

To confirm the results of mRNA-seq associated with IFN responses, we selected six genes from the IFN response genes and evaluated their expression by RT-qPCR. Upon *SP110* and/or *SP140* knockdown, all the selected IFN response genes were significantly downregulated in infected macrophages compared with those in control macrophages (Fig. 6B and C). We further investigated the secretion of CXCL10 in *M. tuberculosis*-infected macrophages with *SP110* and/or *SP140* knockdown (Fig. 7). ELISA revealed that *SP110* and/or *SP140* knockdown significantly reduced the secretion of CXCL10 in *M. tuberculosis*-infected macrophages. These data support the results of mRNA-seq.

## DISCUSSION

Susceptibility to *M. tuberculosis* infection in C3HeB/FeJ mice has been determined by the *Sst1* locus (13). Within the *Sst1* locus, *Sp110* was initially identified as the gene repressed in *Sst1*-congenic mice (15). Several studies have found that single nucleotide polymorphisms (SNPs) in human *SP110* are associated with susceptibility to TB but with no consistent patterns (22–24). Recently, *Sp140* was demonstrated to be the genetic determinant of host susceptibility to *M. tuberculosis* infection in *Sst1*-congenic mice (16). *Sp110*-knockout mice exhibited unaltered bacterial burdens and survival rates compared with those in wild-type mice upon *M. tuberculosis* infection. However, *Sp140*-knockout mice were shown to be more susceptible to *M. tuberculosis* infection. The infected lungs from *Sp140*-knockout mice or *Sst1*-congenic mice demonstrated the upregulation of type I IFNs and their response genes. In this study, we also found that BMMs from C3HeB/FeJ mice induced stronger expression of inflammatory response genes compared with those from C3H/HeN when infected with *M. tuberculosis* (Fig. 3).

Both *SP110* and *SP140* are associated with human immunological diseases (17). Loss-of-function mutations in *SP110* are associated with the veno-occulusive disease with immunodeficiency (25–27). Genome-wide association studies (GWASs) have demonstrated that SNPs in *SP140* are associated with Crohn’s disease (CD) (28, 29), multiple sclerosis (30), and chronic lymphocytic leukemia (31). Previous studies have demonstrated that *SP140*-knockdown human macrophages or macrophages derived from patients with CD who are homozygous for *SP140* SNPs exhibited impaired expression of inflammatory genes, including IFN response genes, upon lipopolysaccharide stimulation (20, 32). These findings supported our results indicating that the induction of inflammatory response genes was impaired in human *SP110*- and/or *SP140*-knockdown THP-1 macrophages when compared with that in control macrophages upon *M. tuberculosis* infection (Fig. 5), which suggests that the regulatory modes of human *SP110* and *SP140* in gene expression associated with inflammatory responses are opposite to those in *M. tuberculosis*-infected mouse macrophages (Fig. 3). Although all functional protein domains are highly conserved, only approximately 45% of amino acids are identical between human and mouse homologs of SP110 and SP140 (17). This lower degree of amino acid sequence identity may explain the functional differences in the gene regulation of SP110 and SP140 between humans and mice.

The expression of pro-inflammatory cytokines, including TNF-α and IFN-γ, is crucial for controlling the burden of *M. tuberculosis* in infected organisms. However, excessive expression of these cytokines leads to cytotoxicity and a loss of control over infected bacilli (33). Prolonged high inflammation would cause necrosis in infected macrophages and recruit additional immune cells, leading to the development of necrotizing granulomas during *M. tuberculosis* infection. We speculate that a high inflammatory state induced by *M. tuberculosis* infection is attributed to the persistent expression of *SP110* and *SP140* in human macrophages, whereas in C3HeB/FeJ macrophages, it is caused by their repressed expression.

M1- and M2-polarized macrophages predominantly utilize aerobic glycolysis and mitochondrial oxidative phosphorylation for ATP generation, respectively (34). Genes associated with oxidative phosphorylation were downregulated upon *M. tuberculosis* infection in macrophages, as described previously (35); however, in *SP110*-knockdown macrophages, they were upregulated during the infection (Fig. 4; Fig. S5), indicating that *SP110* negatively regulates the gene expression associated with oxidative phosphorylation during *M. tuberculosis* infection. Glycolysis and oxidative phosphorylation are also associated with proinflammatory and anti-inflammatory cytokine production to arrest or support the intracellular proliferation of infected *M. tuberculosis* in macrophages, respectively (36). Although we observed no differences in the intracellular proliferation of *M. tuberculosis* and the cell viability of infected macrophages between control and *SP110-* and/or *SP140-*knockdown macrophages (Fig. 2), their knockdown may have an adverse effect on the control of infected *M. tuberculosis* in human macrophages. Considering the impaired expression of type I IFN response genes in *SP110*- and/or *SP140*-knockdown macrophages, the bactericidal activity and induction of cell death by *M. tuberculosis* infection *via* the type I IFN pathway (37) in their knockdown macrophages might be comparable to those in control macrophages.

We found that expression of genes related to oxidative phosphorylation is upregulated by *SP110* depletion in *M. tuberculosis*-infected macrophages (Fig. 4; Fig. S5), whereas ATP concentration in infected macrophages did not change (Fig. S4). Considering that cell viability and growth rate were not changed by *SP110* and/or *SP140* knockdown at 24 and 48 h p.i. (Fig. 2), ATP concentration in macrophages would not be affected by the depletion of *SP110* and/or *SP140* in the early infection period. Since ATP is produced by several pathways other than oxidative phosphorylation, further analysis is needed to determine whether upregulation of gene expression related to oxidative phosphorylation by *SP110* knockdown affects ATP levels.

In this study, we employed siRNA molecules to reduce the gene expression of *SP110* and/or *SP140*. We confirmed that the expression of these genes was significantly decreased at 24 and 48 h p.i. in *M. tuberculosis*-infected macrophages with their knockdown (Fig. S3). However, we must consider the off-target effects of siRNA treatment. Since *SP110* and *SP140* share approximately 55.4% identical nucleotide sequences and have multiple isoforms (17), there is a possibility that siRNA sequences targeting *SP110* or *SP140* also target the other gene. We first used two different molecules targeting *SP140* and found that one of the molecules decreased the gene expression of both *SP110* and *SP140* (Fig. S1). Furthermore, the depletion effect on gene expression does not last long after siRNA transfection. We confirmed that the expression of the target genes for siRNA molecules in THP-1 macrophages increased to 30%‒70% at 5 days post-transfection, corresponding to 3 days p.i. (data not shown). This suggests that we could not study the effect of gene depletion by siRNA treatment over a long infection period. CRISPR-Cas9-mediated gene knockouts of *SP110* and/or *SP140* would allow us to overcome these limitations. Further analysis is required to investigate the precise function of *SP110* and *SP140* in regulating gene expression in *M. tuberculosis*-infected human macrophages.

In conclusion, we observed that *SP110* and *SP140* positively regulate the expression of genes associated with inflammatory responses, including IFN response genes, in human macrophages upon *M. tuberculosis* infection. The results of this study will provide novel insight into the immune responses in human macrophages during *M. tuberculosis* infection and the mechanisms involved in the development of necrotizing granulomas in patients with TB.

## MATERIALS AND METHODS

### Mice

C3HeB/FeJ and C3H/HeN mice were purchased from Jackson Laboratory and Japan SLC, respectively. C3HeB/FeJ mice were maintained in a filtered-air laminar-flow cabinet and provided sterile bedding, water, and mouse chow in the animal facility of the RIT. Specific pathogen-free status was verified by testing sentinel mice housed within the colony.

### Cells

THP-1 cells were obtained from RIKEN BRC and cultured in RPMI-1640 medium (Sigma-Aldrich) supplemented with 10% fetal bovine serum (FBS, Nichirei Bioscience), 100 U/mL penicillin, and 100 µg/mL streptomycin (complete medium). For activation, THP-1 cells (1 × 10^5^ cell/mL) were seeded in a complete medium containing 10 ng/mL phorbol myristate acetate (PMA, Sigma-Aldrich) in 6- or 96-well collagen-coated tissue culture plates (AGC Techno Glass) and incubated for 24 h. Adherent THP-1 cells were washed twice with a complete medium and then incubated in a complete medium without antibiotics for an additional 48 h.

Murine BMMs were differentiated from bone marrow cells as described previously (38). Briefly, bone marrow cells were cultured in DMEM (Sigma-Aldrich) supplemented with 10% L929-conditioned medium, 10% FBS, 100 U/mL penicillin, and 100 µg/mL streptomycin. For 7 days, the cultured BMMs were found to be >95% CD11b-positive. Subsequently, the BMMs were incubated in DMEM supplemented with 10% FBS.

### RNA interference

Small interfering RNA (siRNA) molecules targeting *SP110* and *SP140* were synthesized by Sigma-Aldrich and Dharmacon, respectively. The siRNA sequences are listed in Table S1. Mission siRNA Universal Negative Control (Sigma-Aldrich) was used as the control. Transfection of PMA-stimulated THP-1 cells with siRNA molecules was performed using Lipofectamine RNAiMAX (Thermo Fisher Scientific) according to the manufacturer’s instructions. The lipid–RNA complexes were added at the wash step after PMA activation. After 48 h of transfection, the medium was replaced with a complete medium without antibiotics, followed by subsequent experiments.

### *M. tuberculosis* infection

*M. tuberculosis* strain H37Rv was grown to the mid-logarithmic phase in 7H9 medium supplemented with Middlebrook ADC (BD Bioscience), 0.2% casamino acid, and 0.05% Tween 80 (39) at 37°C. Cell suspensions for infection were prepared as described previously (40) to remove cell aggregates and clumps (41). Briefly, the bacterial cultures were filtered using 5 µm pore size filters, and aliquots of the filtered suspensions were stored at −80°C until use. The bacterial numbers of the stocks were determined by colony-forming unit (CFU) assays using 7H10 agar medium supplemented with 10% Middlebrook OADC (BD Bioscience) and 0.5% glycerol (7H10 agar plate) at 37°C.

Infection with *M. tuberculosis* was performed as described previously (18). Briefly, PMA-stimulated THP-1 cells or BMMs were infected with *M. tuberculosis* at a multiplicity of infection of one in complete medium without antibiotics for 24 or 48 h or DMEM supplemented with 10% FBS for 24 h, respectively.

### Reverse transcription-quantitative PCR

Total RNA was extracted using RNeasy Mini Kit (Qiagen), and reverse transcriptase reaction was performed using Prime Script Reverse Transcriptase (Takara). Subsequently, RT-qPCR was performed using TaqMan Universal Master Mix (Thermo Fisher Scientific). Table S2 lists the primers and probes used in this study. The minus threshold cycle (Ct) value of target genes normalized to that of *GAPDH* (−dCt) was calculated.

### Immunoblot analysis and ELISA

Immunoblot analysis was performed as described previously (18, 42). Briefly, 15 µg of extracted protein was subjected to SDS-PAGE, followed by immunoblot analysis using anti-SP110 (300 dilution, Sigma-Aldrich, HPA047036) or anti-GAPDH (1000 dilution, MBL, M171-3) antibody.

The concentration of secreted CXCL-10 was measured by a Human CXCL10/IP-10 Quantikine ELISA kit (R & D systems). The culture media from *M. tuberculosis*-infected macrophages were collected at 24 h p.i., followed by filtration with a 0.45-µm pore size filter (Toyo Roshi Kaisha).

### Number of intracellular bacilli and viability and growth of macrophages

To determine the number of intracellular bacilli, *M. tuberculosis*-infected PMA-stimulated THP-1 cells were washed twice with PBS at 24 or 48 h post-infection (p.i.) and then harvested using 0.1% SDS in PBS. Samples were serially diluted with sterile water and inoculated onto 7H10 agar plates.

For evaluating the cell viability of *M. tuberculosis*-infected macrophages, infected macrophages in six-well plates were washed twice with PBS at 24 or 48 h p.i. and stained with LIVE/DEAD Fixable Green dye (Thermo Fisher Scientific) for 30 min at room temperature. Stained cells were washed with PBS and then fixed with 10% formalin solution for more than 24 h at 4°C. After fixation, the cells were washed with PBS and incubated with 5 mM EDTA in PBS for 30 min at room temperature, followed by collection. The collected cells were suspended with FACS buffer (2 mM EDTA and 2.5% FBS in PBS) and analyzed using BD FACSLyric flow cytometer (BD Bioscience).

To evaluate the cell growth of *M. tuberculosis*-infected macrophages, infected macrophages in 96-well plates were incubated with MTT solution for 2 h at 37°C (Nakalai tesque) according to the manufacturer’s instructions. The macrophages were lysed with the lysis buffer and subsequently incubated for an additional 24 h at 37°C. Optical absorbances at 570 and 655 nm were measured using a microplate reader. The absorbance at 570 nm of the samples was corrected by both the absorbance at 570 nm of the blank and the absorbance at 655 nm of the samples.

### mRNA-seq

The quality and quantity of isolated RNA were evaluated using a Qubit Fluorometer (Thermo Fisher Scientific) and RNA ScreenTape on the 4150 TapeStation system (Agilent Technology). Briefly, 1 µg of total RNA with an RNA integrity number of >7.0 was used to construct a cDNA library using NEBNext Poly(A) mRNA Magnetic Isolation Module (New England Biolabs) and NEBNext Ultra II Directional RNA Library Prep Kit for Illumina (New England Biolabs). All cDNA libraries were checked for quality and quantity using a Qubit Fluorometer and DNA high-sensitivity Screen Tape on the 4150 TapeStation system. The libraries were sequenced on Illumina NextSeq 1000 to generate approximately 20 million 50-base-long paired-end reads per library.

### Data processing

RNA-seq data were processed as described previously (18, 43) with slight modification. Briefly, raw reads were processed using Trim Galore (version 0.6.7) for read-quality trimming (https://github.com/FelixKrueger/TrimGalore). The processed reads were then aligned with STAR (version 2.7.10b) (44) against the human genome hg38. Gene counts were determined using featureCounts (version 2.0.1) (45). Differential gene expression analysis was conducted using edgeR (version 3.36.0) (46) with generalized linear models and quasi-likelihood tests (47). DEGs were identified using the cutoff *P* value with a false discovery rate of 0.05 provided by edgeR (Table S3.1 and Table S3.2). The DEGs were further analyzed for Gene Ontology (GO) enrichment analysis using clusterProfiler (version 4.2.2) to visualize enriched biological process terms (GOBP) (48). GSEA (49) was conducted based on transcript per million data with hallmark gene sets collected from the Molecular Signature Database (MSigDB). Significantly enriched gene sets were determined using the cutoff of the adjusted *P* values at 0.25.

Heatmap, hierarchical cluster analysis, ANOVA, Tukey–Kramer multiple comparison test, and Dunnett’s multiple comparison test were performed using R version 4.1.2.

## Data Availability

Raw sequence data have been deposited in the DRA database under the accession number DRA017561 and DRA017562.

## SUPPLEMENTAL MATERIAL

Figure S1 spectrum.00101-24-s0001.tiff Off-target effect of siRNA targeting *SP140* on *SP110* expression.

Figure S2 spectrum.00101-24-s0002.tiff Immunoblot analysis of *SP110* in macrophages with *SP110* and/or *SP140* knockdown.

Figure S3 spectrum.00101-24-s0003.tiff Knockdown efficacy of *SP110* and/or *SP140* in *M. tuberculosis*-infected macrophages.

Figure S4 spectrum.00101-24-s0004.tiff ATP concentration in THP-1 macrophages with knockdown of *SP110* and/or *SP140*.

Figure S5 spectrum.00101-24-s0005.tiff Heatmap visualization of the expression of genes related to oxidative phosphorylation.

Figure S6 spectrum.00101-24-s0006.tiff Genome-wide analysis of the gene expression.

Table S1 spectrum.00101-24-s0007.xlsx siRNA targeting genes and primers.

Table S2 spectrum.00101-24-s0008.xlsx Target genes and the primers used for RT-qPCR.

Table S3.1 spectrum.00101-24-s0009.xlsx RNA-seq data analyzed using edgeR.

Table S3.2 spectrum.00101-24-s0010.xlsx RNA-seq data analyzed using edgeR.

ASM does not own the copyrights to Supplemental Material that may be linked to, or accessed through, an article. The authors have granted ASM a non-exclusive, world-wide license to publish the Supplemental Material files. Please contact the corresponding author directly for reuse.
