# Supplementary figures and images for: Transcriptional regulators SP110 and SP140 modulate inflammatory response genes in Mycobacterium tuberculosis-infected human macrophages
Source: Microbiol Spectr. 2024 Aug 20;12(10):e00101-24. doi: 10.1128/spectrum.00101-24 (PMC11448263; doi:10.1128/spectrum.00101-24)

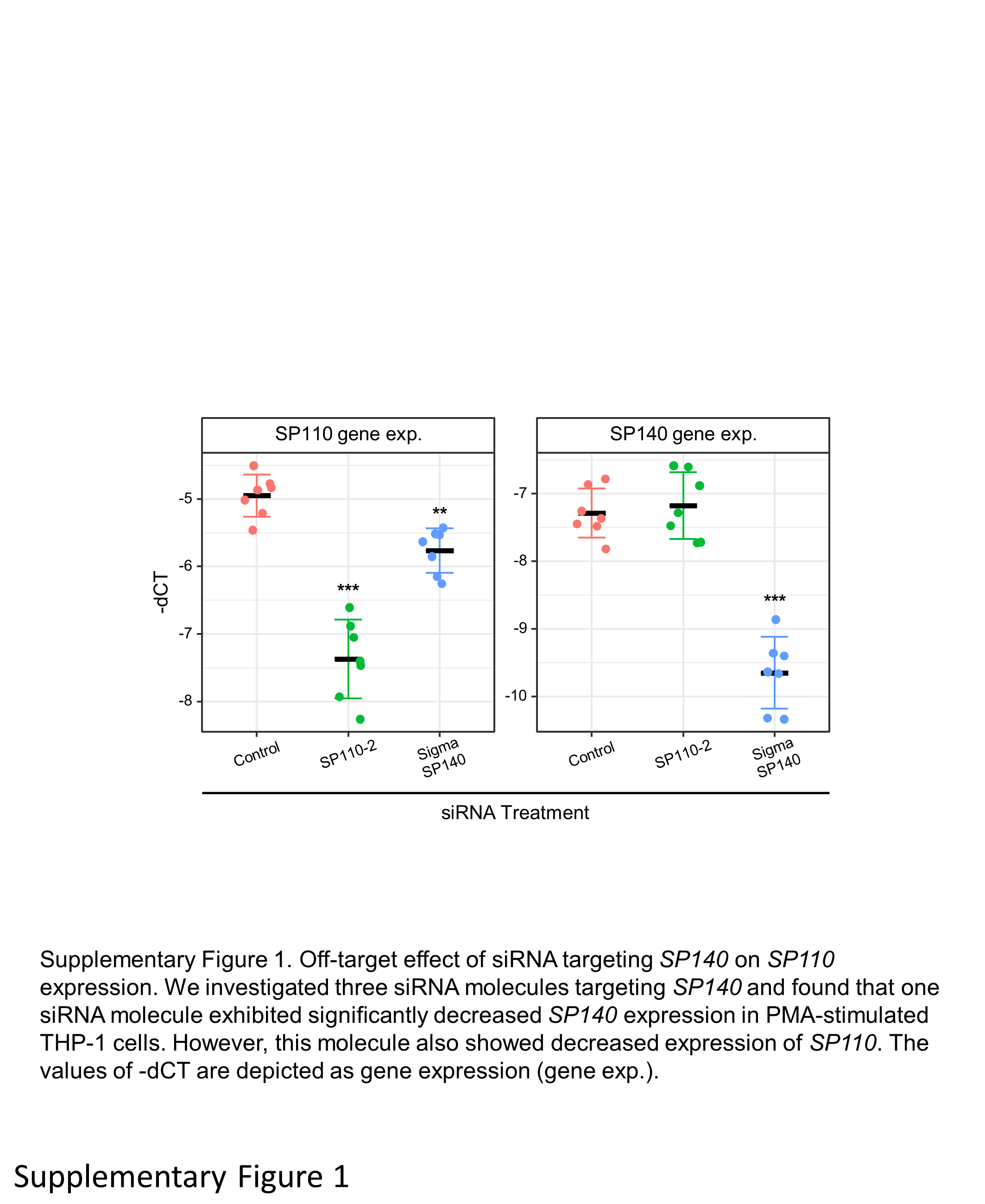

Supplement: Figure S1 — Off-target effect of siRNA targeting SP140 on SP110 expression. [file spectrum.00101-24-s0001.tiff]

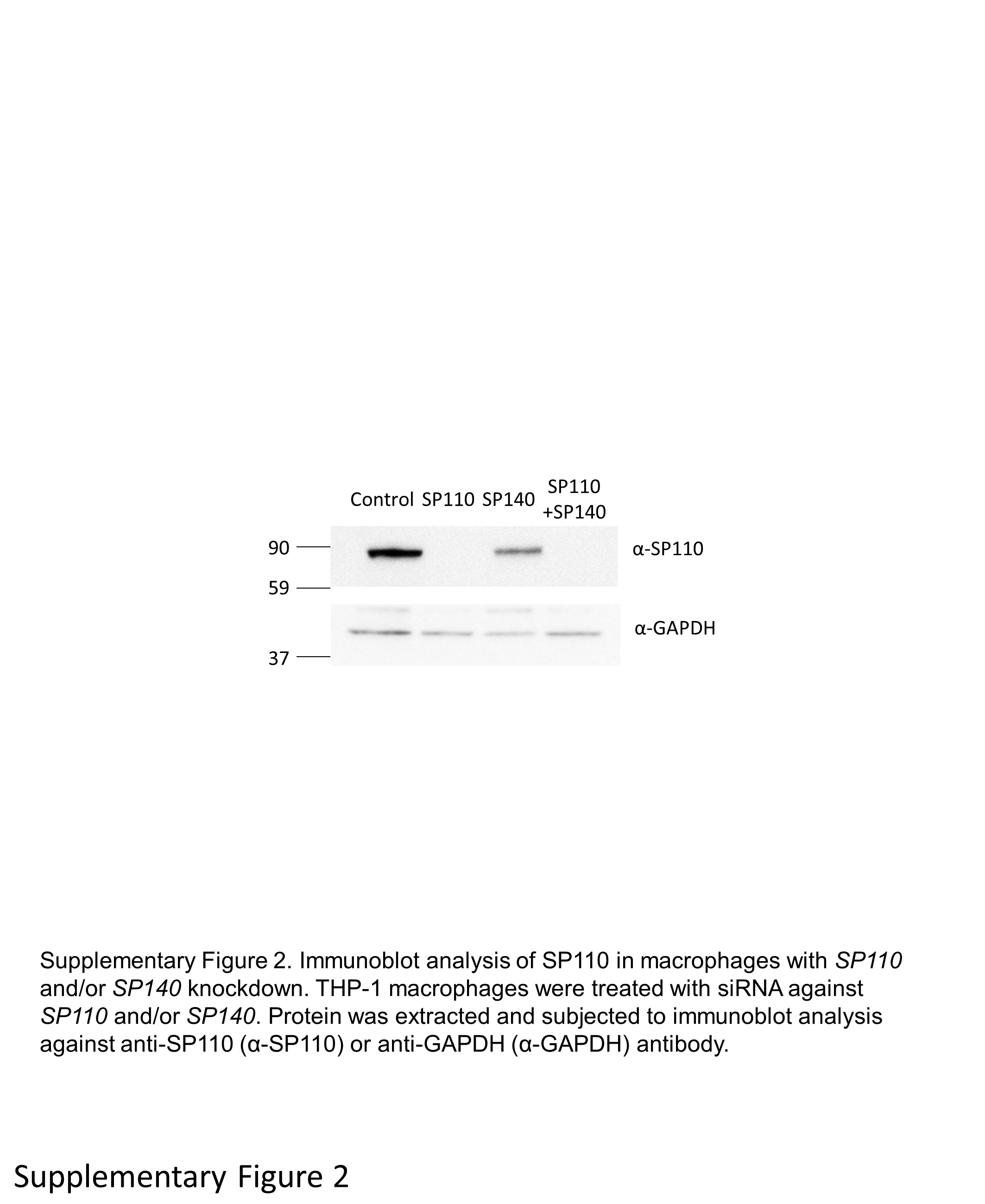

Supplement: Figure S2 — Immunoblot analysis of SP110 in macrophages with SP110 and/or SP140 knockdown. [file spectrum.00101-24-s0002.tiff]

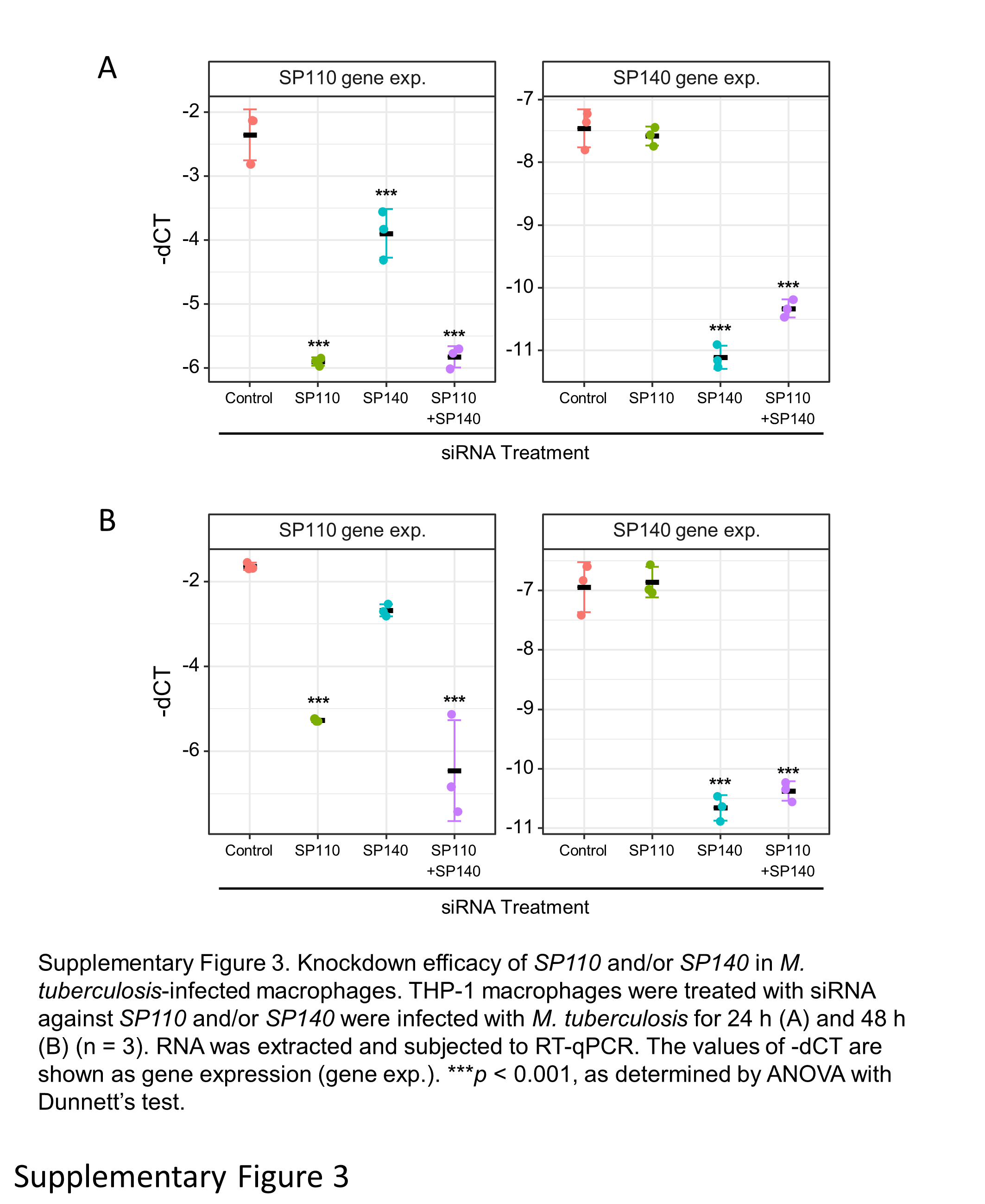

Supplement: Figure S3 — Knockdown efficacy of SP110 and/or SP140 in M. tuberculosis-infected macrophages. [file spectrum.00101-24-s0003.tiff]

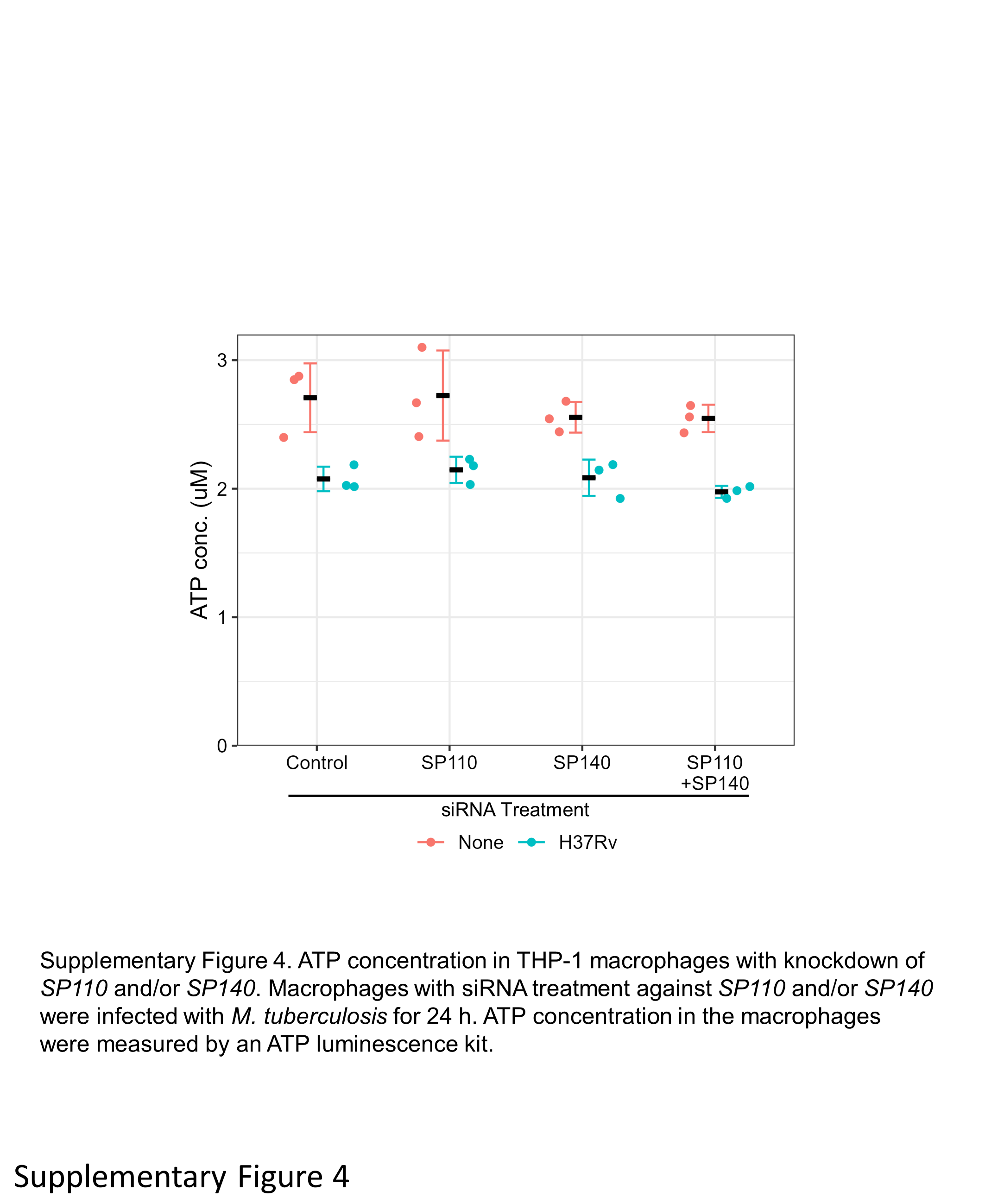

Supplement: Figure S4 — ATP concentration in THP-1 macrophages with knockdown of SP110 and/or SP140. [file spectrum.00101-24-s0004.tiff]

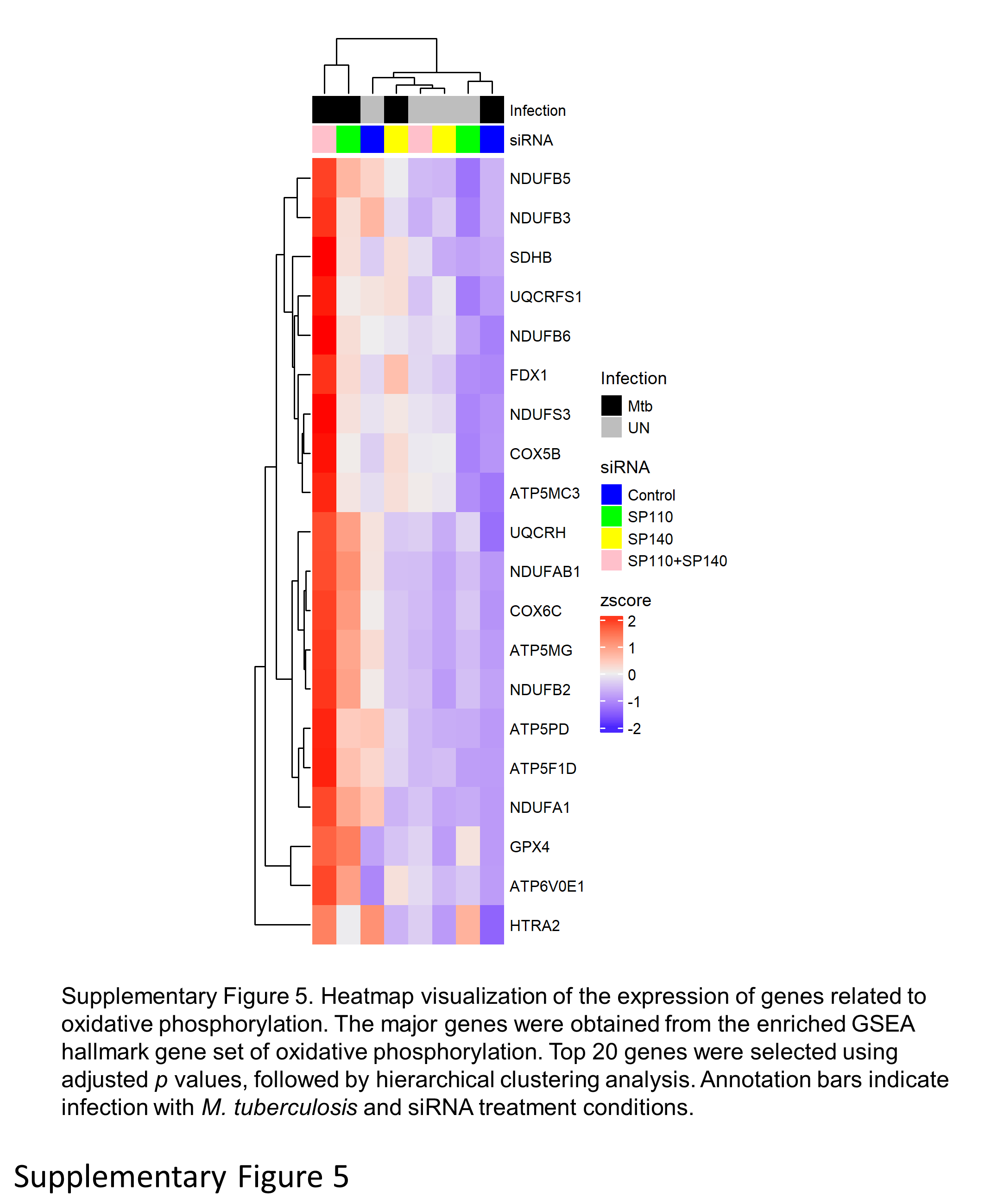

Supplement: Figure S5 — Heatmap visualization of the expression of genes related to oxidative phosphorylation. [file spectrum.00101-24-s0005.tiff]

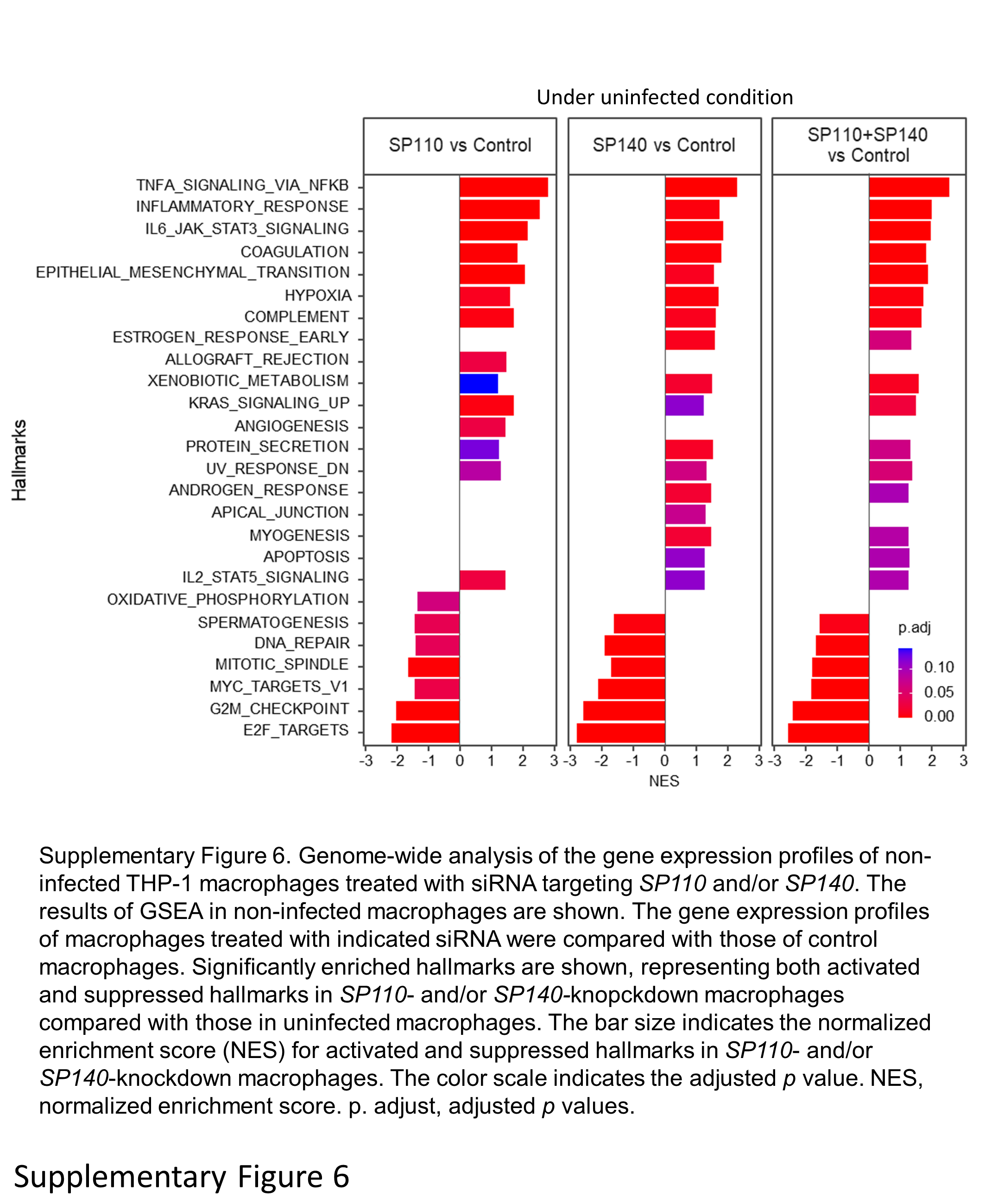

Supplement: Figure S6 — Genome-wide analysis of the gene expression. [file spectrum.00101-24-s0006.tiff]
